# Training‐Induced Neural Enhancement of Novel Song Learning in Chronic Aphasia: EEG Study

**DOI:** 10.1111/nyas.70087

**Published:** 2025-10-13

**Authors:** Emma Oksanen, Emmi Pentikäinen, Anni Pitkäniemi, Sini‐Tuuli Siponkoski, Sari Laitinen, Essi‐Reetta Särkämö, Aleksi J. Sihvonen, Tommi Makkonen, Jaakko Kauramäki, Teppo Särkämö

**Affiliations:** ^1^ Cognitive Brain Research Unit & Centre of Excellence in Music, Mind, Body and Brain, Department of Psychology, Faculty of Medicine University of Helsinki Helsinki Finland; ^2^ Espoo Hospital Espoo Finland; ^3^ Private Choir Conductor Vantaa Finland; ^4^ Department of Neurology Helsinki University Hospital Helsinki Finland; ^5^ Queensland Aphasia Research Centre University of Queensland Brisbane Australia

**Keywords:** aphasia, auditory processing, electroencephalography, learning, mismatch negativity, MMN, singing

## Abstract

While singing‐based rehabilitation can aid language recovery in aphasia, little is currently known about how persons with aphasia (PWA) are able to learn new songs. Using data from a previous crossover randomized controlled trial, this electroencephalography (EEG) study explored the effects of a 16‐week multicomponent singing intervention on neural learning of novel song material in 24 PWA, reflected by the mismatch negativity (MMN) response, and its correlation with changes in communication and verbal learning. In pre‐ and postintervention EEG measurements, PWA participants listened to modulated versions of two novel songs (trained vs. untrained during intervention) using a passive oddball design with pitch, phoneme, and duration deviants embedded in the melody. Significant changes in MMN were observed for the trained versus untrained songs from pre‐ to postintervention for phoneme and frequency deviants. Especially for phoneme deviants, the MMN amplitude in left frontotemporal areas decreased for the trained song but increased for the untrained song. These findings suggest that singing intervention can induce neural learning of song material in PWA.

## Graphical abstract

We examined how a singing intervention affects the ability of persons with aphasia to learn new songs. Behavioral and EEG results showed that the singing intervention enhanced verbal production of trained song lyrics, coupled with reduced mismatch negativity (MMN) responses to phoneme and frequency changes. These findings suggest that a singing intervention can induce neural learning in aphasia.

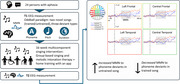

## 1 Introduction

Perceiving and decoding our complex auditory environment and being able to respond to it are essential skills in everyday life and in verbal communication. At the perceptual level, incoming auditory information is segregated into individual components, based on temporal and spectral cues and recognition of learned patterns [1]. As the patterns of the auditory environment are extracted, mostly by implicit processes, the brain can formulate a probabilistic model of the auditory environment, which can produce predictions about the forthcoming auditory events [2]. If incoming auditory events do not match these predictions, a change detection process is elicited [2, 3]. This can be observed as a change in the event‐related electrical activity of the brain, known as the mismatch negativity (MMN) response [4, 5].

Typically observed in electroencephalography (EEG) measurements, MMN is a negative event‐related potential (ERP) component, which peaks between 150 and 200 ms after the onset of a deviant stimulus in a continuous stream of standard stimuli [5, 6]. In the auditory domain, it can be elicited by deviant phonemes [7, 8] and complex tones (musical sounds) [9, 10], among other stimuli. While the source of the MMN is generally located in the superior temporal and prefrontal cortices [11], its exact location and laterality can differ depending on the modality of the sounds [12], with more bilateral responses to musical stimuli and more left‐lateralized responses to linguistic stimuli, reflecting the left hemisphere dominance of language processing [8, 10, 13, 14]. MMN can be elicited in the absence of attention [11] and even when the participants do not consciously detect the deviant stimuli [15], making it ideal for measuring auditory‐cognitive processing in language disturbances such as aphasia.

Aphasia, a language disorder caused by left hemisphere damage and affecting up to 40% of stroke survivors [16, 17, 18], has been linked to abnormalities in auditory‐cognitive processing. Previous studies have shown that persons with aphasia (PWA) exhibit absent, diminished, or delayed MMN responses, especially to linguistic stimuli, such as vowels or consonants [19, 20, 21, 22, 23, 24, 25, 26, 27, 28]. Additionally, the topographical distribution of MMN to linguistic stimuli tends to show less lateralization toward the left hemisphere in PWA [21, 28], most likely owing to left frontotemporal damage in aphasia. Conversely, PWA seem to demonstrate relatively normal MMN responses to deviants in simple tones, such as deviating pitch, although reduced MMN responses to complex harmonic tones have been observed [23, 25, 26]. Research on the effects of rehabilitation on the MMN response in PWA is limited, but a few studies have reported enhancement of the linguistic MMN response in the left hemisphere following high‐intensity speech and language therapy (4 weeks, 3.5 h on 3 days per week) [29] and constraint‐induced aphasia therapy (2 weeks, 3–4 h on 5 days per week) [30], coupled with behavioral improvement in clinical language tests. These intensive aphasia therapy methods involve functional verbal interaction through structured tasks and language games, with limited use of nonverbal communication (e.g., gestures).

Since the early 18th century case studies [31], it has been reported that some PWA have relatively well spared capability to produce words through singing, despite severe difficulties in speech production [32, 33]. This phenomenon may be attributed to the findings that singing relies partly on distinct neural networks compared to speaking [34, 35, 36]. Preservation of the left middle temporal gyrus (MTG), one of the areas that have shown functional specificity to singing in the healthy brain [35], and the ventral white matter tracts connecting it to prefrontal areas, have been associated with better sung versus spoken word production in PWA [37, 38].

The spared singing capabilities of PWA have been harnessed in the form of music‐based interventions, such as melodic intonation therapy (MIT), which have shown promise in enhancing communication outcomes in PWA, including increased verbal output and improved daily life communication [39, 40, 41]. MIT is a structured speech therapy approach that uses melodic and rhythmic elements of speech to facilitate verbal expression, progressing from intoned phrases to more natural, speech‐like production. While traditionally individually delivered therapies such as MIT are effective, their one‐on‐one format may limit opportunities for social interaction during rehabilitation. In contrast, group‐based methods such as choir singing interventions offer a social dimension, which may improve a sense of belonging and reduce social isolation. Previous studies have shown that group‐based singing interventions can improve communication outcomes, language recovery, and social well‐being of PWA [42, 43, 44] coupled with increased gray matter volume in the left inferior frontal cortex and enhanced structural connectivity in multiple bilateral white matter pathways [45].

Currently, little is known about the effects of singing‐based interventions on auditory‐cognitive processing and learning of novel song material in PWA. Using longitudinal EEG data collected in the Helsinki Singing in Aphasia Study [42], this study set out to explore the effects of a multicomponent singing intervention in PWA on the neural processing of linguistic (phoneme), spectral (frequency), and temporal (duration) changes embedded in novel songs either trained or not trained during the intervention, as measured with the MMN response. Previous results of the Helsinki Singing in Aphasia Study have shown positive effects of the singing intervention on everyday communication and responsive speech production (repetition and naming) [42] as well as on structural neuroplasticity in left‐hemispheric frontal language areas and bilateral white matter pathways associated with speech production [45] in PWA. To extend these findings, this study sought to determine: (i) if the intervention can also induce neural learning of novel songs in PWA, (ii) whether the effect is specific to the trained song material or also generalized to untrained song material, and (iii) whether it correlates with behavioral changes in communication abilities and the verbal learning of the song lyrics.

## 2 Methods

### 2.1 Participants

A subsample of 34 PWA from the Helsinki Singing in Aphasia Study [42] participated to the present EEG study. The data of 10 participants were excluded due to insufficient EEG data quality or missing measurements (see “EEG recording and data preprocessing” for details). Demographic and clinical information of the remaining 24 participants are presented in Table 1. The participants were recruited through patient organizations and speech therapists from the Helsinki metropolitan area during 2017–2019 and interviewed by the recruiting psychologists of the study to assess their eligibility for the study. Inclusion criteria were: (1) aged over 18, (2) Finnish‐speaking, (3) time since stroke or injury over 6 months, (4) at least mild aphasia (Boston Diagnostic Aphasia Examination [BDAE] Aphasia Severity Rating Scale [46] score ≤4, preliminary assessment based on recruitment interview), (5) no subjective hearing deficit, (6) cognitive ability to give informed consent, (7) no neurological/psychiatric comorbidity or substance abuse, and (8) ability to produce vocal sound through singing or humming. The study was approved by the Ethics Committee of the Helsinki and Uusimaa Hospital District, and written informed consent was obtained from all participants.

**TABLE 1:** Background information.

	Participants included in the analysis after preprocessing (*N* = 24)
Age (mean [SD])	63.6 (9.8)
Sex (female/male)	11/13
Handedness (right/left)	21/3
Etiology of injury (ischemic/hemorrhagic/both)	14/7/3
Time since injury (months) (mean [SD])	85.2 (83.0)
Aphasia severity^a^ (mean [SD])	3.0 (1.4)
Education level^b^ (mean [SD])	3.1 (1.3)

^a^ Aphasia severity according to the Boston Diagnostic Aphasia Examination/Aphasia Severity Rating Scale score: range 0 (no usable speech) to 5 (minimal discernible speech handicaps).

^b^ Education level according to the UNESCO International Standard Classification of Education: range 1 (primary education) to 6 (doctoral or equivalent level).

### 2.2 Study Design

**FIGURE 1:**
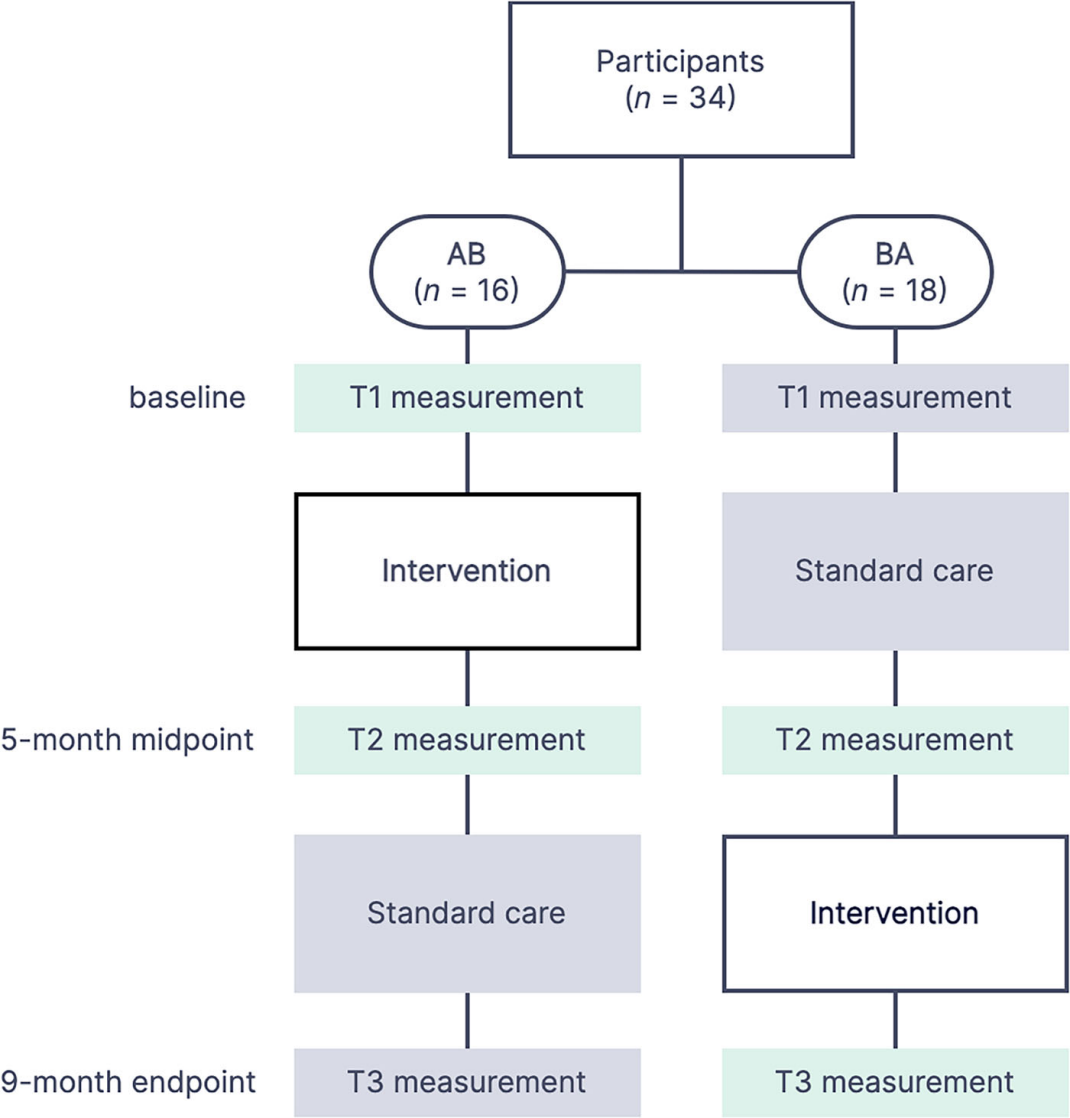
Flowchart of the study design and participant allocation to groups AB and BA, and number of participants included in the analysis. Standard care included standard speech and language therapy, neuropsychological rehabilitation, and physical/occupational therapy provided by public health care.

All participants took part in a 16‐week multicomponent singing intervention. To ensure access to intervention for all participants, the study was implemented using a crossover design where participants received the intervention either during the first (AB group) or second (BA group) half of the study period (Figure 1). Behavioral and EEG measurements were performed on all participants before (T1) and after (T2) the intervention.

### 2.3 Multicomponent Singing Intervention

The 16‐week intervention included weekly group sessions lasting 1.5 h each (total 24 h) and home training aimed at three sessions per week, 30 min each (total 24 h). On average, the participants attended 90.1% (SD = 14.0) of group sessions and completed 11.9 h (SD = 9.8) of home training. The intervention was administered to all groups by the same two‐person team comprising a choir conductor and singing teacher (E.‐R.S.) and a music therapist (S.L.).

Group training sessions consisted of 60 min of singing training and 30 min of group‐based MIT. Singing training included breathing exercises, vocal warm‐ups (20 min/session), and group singing (40 min/session) during which participants trained the production of the song repertoire comprising 10 songs (mainly familiar songs and a novel song, see below). Group‐based MIT focused on singing‐based training of formulaic speech phrases, following traditional MIT methods such as simultaneous tapping and structured progression from modeling to repetition [47]. Home training utilized a tablet‐based app named Singalonger, developed in collaboration with a Finnish company Outloud Oy (Tampere, Finland, https://outloud.fi/), featuring songs from the group singing repertoire. Participants could train the songs with three kinds of training aids: instrumental or sung auditory model, karaoke‐type lyrics, or video of mouth movements. The app automatically recorded participants’ singing, analyzed the pitch and duration of notes, and provided online feedback (star‐rating) for motivation. More detailed description of the intervention can be found in Siponkoski et al. [42].

**FIGURE 2:**
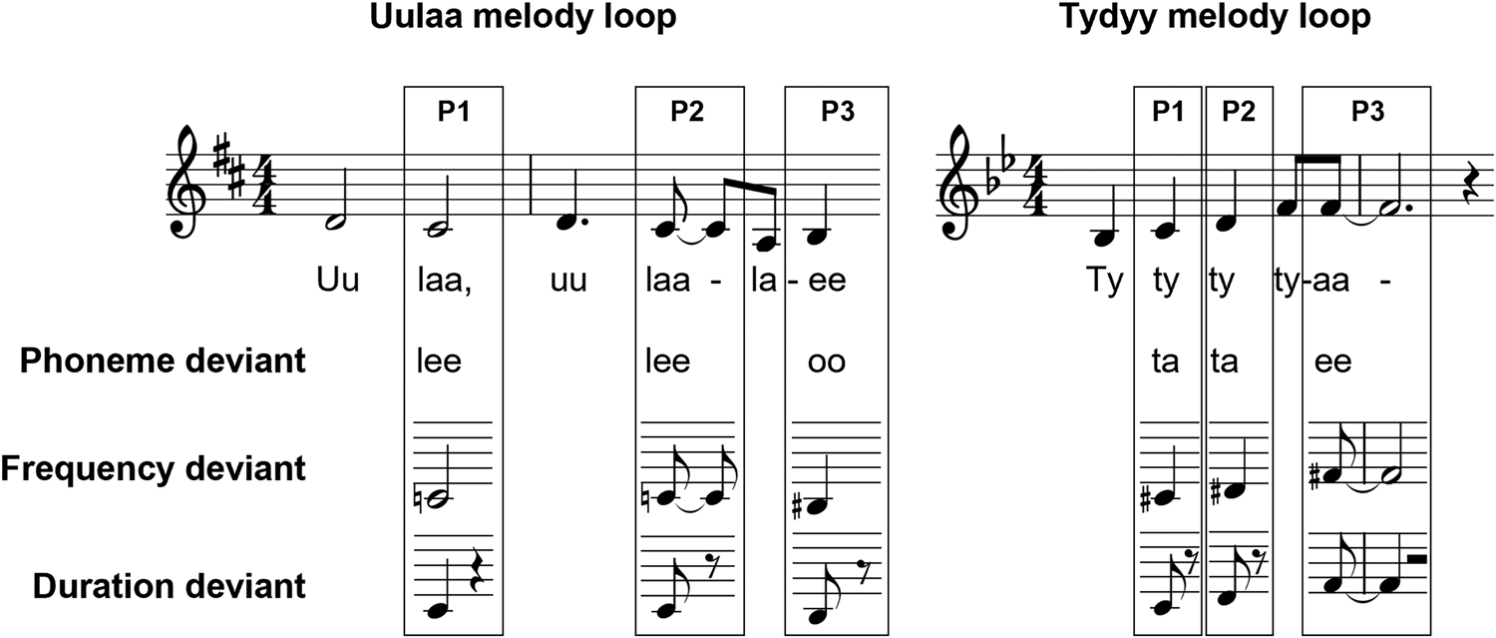
Chorus parts of songs “Uulaa” (left) and “Tydyy” (right) with phoneme, frequency, and duration deviants.

During the intervention, the participants trained one of two new songs, composed for the purpose of this study. Group AB trained the song “Uulaa,” and group BA trained the song “Tydyy” (score and Finnish lyrics shown in Supporting Information Figure 1). The lyrics of the songs reflected the life experiences of PWA (e.g., adaptation to changes, resilience, hope). The songs were trained regularly during both group and home training sessions over the 16 weeks as part of the intervention. The chorus parts of the songs (Figure 2) served as stimuli in the EEG experiment.

### 2.4 Experimental Conditions and Stimuli

During the EEG experiment performed at T1 and T2, participants heard ongoing sung melody loops of either song “Uulaa” or song “Tydyy,” in two separate blocks. The order of the blocks was randomized and then reversed for the follow‐up (T2) measurements. All the melody loops were sung by the same female singer. Before the actual experiment, hearing thresholds were measured with a portable audiometer Oscilla USB350SP and AudioConsole software (Natus Medical Denmark APS, Taastrup, Denmark) from 11 different frequencies spanning from 250 to 8000 Hz. The melody loops were presented in the EEG lab from speakers (OWI‐202, OWI Inc., Carson, California, USA) about 1 m away from the participant. The intensity of the stimuli was 70 dB for participants with normal hearing (based on the age‐level normative data included in the AudioConsole software), 75 dB if the hearing threshold was ≤10 dB above normal, and 80 dB if it was >10 dB above normal. During the experiment, the participants were asked to ignore the sounds and concentrate on watching a silent movie from a screen. After the experiment, questions about the movie were asked to ensure the participants’ concentration on the movie.

One melody loop of song “Uulaa” comprised six sung syllables, and one melody loop of song “Tydyy” composed of five sung syllables (Figure 2). Here, individual syllables are referred to as stimuli. The experiment was implemented with an oddball design; 90% of the loops included a deviant stimulus in one of three positions (P1–P3, Figure 2), while the stimuli in the other positions remained standard. The position of the deviant was altered in randomized order to keep the timing of the deviants unpredictable to the participant. There were three kinds of deviant stimuli (Figure 2): (i) phoneme deviants with a vowel change in the sung syllable, (ii) frequency deviants with a pitch change of one semitone in the sung syllable (in the direction of the contour of the melody), and (iii) duration deviants with a shortening (50%) in the duration of the sung syllable (followed by same duration lengthening of the subsequent break after the syllable in order not to alter the global rhythmic pattern of the melody). To ensure comparability, only the standard stimuli from the same positions as deviant stimuli were used in the analyses.

The duration of one whole melody loop was 4300 ms and there were 241 melody loops in each block, making the total duration of each block about 17 min. In the analysis, the stimuli from the three positions (P1–P3) of the melody loop were pooled together. Altogether, the melody loops in one block included 72 of each type of deviant stimuli (phoneme/frequency/duration) and 507 standard stimuli from positions P1–P3. The proportion of each type of deviant stimuli in one block was 10%, and the three types of deviants had an equal probability of appearance.

### 2.5 EEG Recording and Data Preprocessing

EEG was recorded with BioSemi Active Two (BioSemi B.V., Amsterdam, the Netherlands) using 64 Ag–AgCl active electrodes, following the standard 10/20 system. Eye movements were monitored with three electro‐oculography electrodes. Two of them were attached to the corners of the eyes, and one under the left eye. Mastoid electrodes were placed behind both ears to be used as reference electrodes later. Data were recorded with DC‐104 Hz bandwidth and sampling rate of 512 Hz.

Raw EEG data were processed with Matlab R2021a (Mathworks Inc., Natick, MA, USA) using EEGLAB version 2022 and in‐house toolbox CBRU plugin version 2.1.4b. First, the data were downsampled to 256 Hz, re‐referenced to the average of the right and the left mastoid channels, and band‐pass filtered (high‐pass filter at 0.5 Hz and low‐pass filter at 30 Hz). Then, the data were separated into epochs and time‐locked to the onset of stimuli, with a 100 ms prestimulus baseline and extending to 500 ms poststimulus onset. Independent component analysis was carried out to identify and remove noise artifacts. Components were visually inspected to identify the ones that included eye blinks and horizontal eye movements. These two components were removed for each participant, if identified. On average, 2.0 (SD = 0.1) components were removed for each participant. After component removal, individual electrodes with inadequate quality data were identified by visual inspection and interpolated. On average, 3.0 (SD = 1.8) channels were interpolated for each participant. Lastly, all epochs that exceeded a threshold of ± 100 µV were removed. In total, 7.0% (SD = 8.3%) of standard trials, 4.8% (SD = 8.0%) of phonemic deviants, 4.9% (SD = 8.4%) of frequency deviants, and 4.3% (SD = 5.4%) of duration deviants were removed for each participant. Participants with insufficient data quality (*n* = 3) or missing data (*n* = 7) at any time points were excluded from the analysis. Due to the characteristics of the patient population, some participants had difficulty staying still throughout the measurement or fully understanding the instructions, which lead to insufficient quality of the EEG data. After preprocessing, 24 participants remained for the analysis.

### 2.6 ERP Formation

**FIGURE 3:**
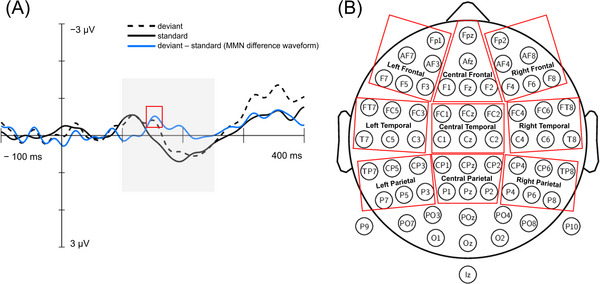
(A) Example of the formation of the MMN difference waveform (blue line), showing the preselected time window of 100–250 ms for the MMN (gray box) and 25 ms around the peak amplitude of the MMN (red box). (B) Nine regions of interest and their corresponding electrodes. MMN, mismatch negativity.

ERP waveforms were created by averaging the remaining data separately for each electrode. The MMN difference waveforms were formed by subtracting the ERP response to the standard stimulus from that of the deviant stimulus. From the channel‐wise difference waveforms, the peak amplitude of the MMN was established by identifying the point of maximum negativity in preselected time window of 100–250 ms (gray box in Figure 3A). Mean amplitudes were then calculated from the 25 ms that surrounded the peak amplitude (±12.5 ms from the peak, red box in Figure 3A). Nine regions of interest (ROI) including frontal, temporal, and parietal regions (Figure 3B) were selected based on previous literature on MMN source localization in healthy [48, 49, 50, 51, 52] and clinical [21, 28, 53] populations. Mean responses of each ROI were used in the statistical analyses.

### 2.7 Outcome Measures for Communication Abilities and Verbal Learning

The outcome measures for communication abilities and verbal learning were constructed based on the variables used in Siponkoski et al. [42], in which a more detailed explanation of the principles underlying their formation is provided. Daily communication was assessed with the Communicative Activity Log (CAL) [54] and the communication subscale of the Stroke Impact Scale 3.0 (SIS) [55] which were both administered to PWAs (self‐report) and family caregivers (FCs, informant‐report) at T1 and T2. For the analysis, a common Communication index was calculated by averaging the percentage scores (score/total × 100) of the PWAs and FCs in the CAL communication total score and SIS communication subscale score (reversed to match the CAL). Automatic and stimulus‐dependent spoken language production was assessed with the Western Aphasia Battery (WAB) [56]. Repetition and Naming indices of WAB were averaged together to form the Responsive speech index, reflecting cued verbal expression ability [57].

To assess the behavioral (verbal) learning of the novel songs, the PWA participants completed a task in which they sung the lyrics of songs Uulaa and Tydyy aloud at T1 and T2. The task was presented using Presentation software (Neurobehavioral Systems, Inc., Berkeley, CA, USA, www.neurobs.com). First, the participants listened to the song through DPA d:fine Dual Earset Omni Tan headset (DPA Microphones, Kokkedal, Denmark), while simultaneously viewing its lyrics on the laptop screen. The lyrics were displayed on a dark background line by line, karaoke‐style. To optimize the pitch for each participant, the songs were presented with a male voice for the male participants, and with a female voice for the female participants. After the passive listening round, the song was presented again with the lyrics on the screen, and the participants were asked to sing along as much as possible. All participants completed the task with both songs, in randomized order. The singing performance was recorded with the microphone in the headset. The performances were then transcribed, and almost correct and fully correct syllables were summed together to form an overall score. As the songs contained different number of syllables, the overall score for each song was converted into percentage for comparability by dividing it by the total number of syllables in the song.

### 2.8 Statistical Analysis

For group AB, song Uulaa was the song that they had trained, while song Tydyy was unfamiliar to them, and vice versa for group BA. For the analysis, the data of the two groups were pooled together due to the small sample size and to control for the potential effect of which song was trained. Therefore, in the analysis there was one group of 24 participants in which every participant had a trained (Uulaa/Tydyy) and an untrained (Tydyy/Uulaa) song.

The data were analyzed using RStudio 4.2.2 [58]. The three deviant types were analyzed separately using linear mixed models (LMMs) to analyze the effects of intervention on the MMN. The models were fitted using the *lme4* package in R [59]. First, a main LMM that included all the nine ROIs was fitted. Previously calculated mean amplitude of the MMN was used as the dependent variable in the model, and independent variables were Time [before (T1)/after (T2) the intervention], Familiarity of the song (trained/untrained), and their interaction. The interaction was included in the model to investigate if the effect of the intervention was generalized or specific for the trained material. To account for the potential variability in the effect of the intervention on the MMN across different ROIs, random intercept and slope were allowed for that interaction, nested within participants. The significance of fixed effects was assessed using *t*‐tests with Satterthwaite's approximation for degrees of freedom. To control the family‐wise error rate in these analyses, a Bonferroni correction was applied, and results with *p* ≤ 0.017 (0.05/3) were considered significant. After the main model, individual LMMs were fitted for each ROI for those MMN responses which showed a significant Time × Familiarity interaction in the main model to see which ROIs carry a significant effect of the intervention. To control the family‐wise error rate in these analyses, a Bonferroni correction was applied, and results with *p* ≤ 0.0056 (0.05/9) were considered significant. Paired *t*‐tests were used for post hoc testing for those ROIs that showed a significant Time × Familiarity interaction in the ROI‐level model. This was done to compare the mean amplitudes of the MMN between the two measurement points (T1 vs. T2). Separate *t*‐tests were performed for the trained and untrained songs, and results with *p* ≤ 0.05 were considered significant.

Pearson correlation coefficient was used to investigate the relationship between the MMN and language performance and verbal learning. The significance of the correlation coefficients was assessed using two‐tailed *t*‐tests. To control the family‐wise error rate in these analyses, a Bonferroni correction was applied, and results with *p* *≤* 0.0016 (0.05/32) were considered significant.

## 3 Results

### 3.1 Training‐Induced Changes in MMN Responses

First, repeated‐measures ANOVAs were performed for each deviant type to determine the effects of Group (AB/BA) and Song (Tydyy/Uulaa) on the MMN amplitudes at T1 (see Table S1), given that the BA group had already heard the songs once at the initial measurement (Measurement I), which could potentially affect the MMN responses at the preintervention time point (T1). There were no significant main effects of Group or Song on the amplitudes of the phoneme, frequency, and duration MMN response, suggesting that the groups and songs were comparable at baseline and that the groups could be pooled together for the main longitudinal analyses.

**FIGURE 4:**
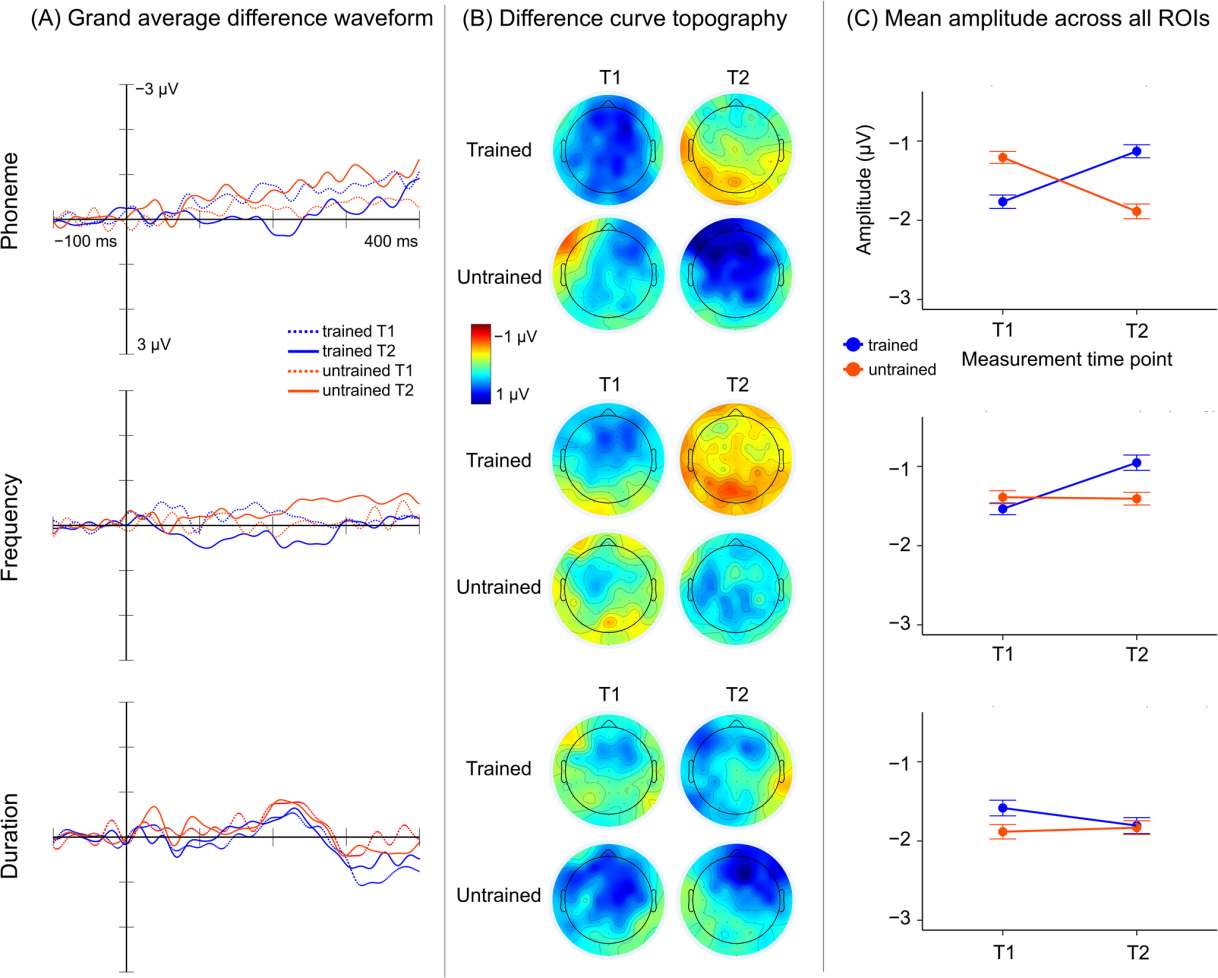
(A) Grand average difference waveform, (B) difference curve topography, and (C) mean amplitude of the MMN response to phoneme, frequency, and duration deviants, across all ROIs. Error bars depict 95% confidence intervals. MMN, mismatch negativity; ROI, regions of interest.

Grand average waveforms, topographies, and mean amplitudes of the MMN response to phoneme, frequency, and duration deviants at T1 and T2 are shown in Figure 4. The LMMs showed significant main effects for Time (T1/T2) in the phoneme MMN response and for Familiarity (trained/untrained) in phoneme and duration MMN responses as well as significant interaction effects between Time and Familiarity in phoneme and frequency MMN responses (Table 2), indicating a training‐dependent change in the amplitude of the MMN. For phoneme deviants, the model estimates suggested a decrease in MMN amplitude for the trained song, and a relative increase for the untrained song. A similar interaction was found for frequency deviants, as MMN amplitude to the trained song showed a model‐estimated decrease over time, while MMN amplitude to the untrained song remained stable.

**TABLE 2:** Fixed effects of the main linear models for each deviant type, and corresponding standard errors, *t* values, and *p* values.

	Phoneme deviants				Frequency deviants				Duration deviants			
Fixed effects	*β*	SE	*t*(df)^c^	*p* ^d^	*β*	SE	*t*(df)^c^	*p* ^d^	*β*	SE	*t*(df)^c^	*p* ^d^
Intercept	−1.62	0.16	−10.39(29)	**< 0.001**	−1.13	0.12	−9.70(44)	**< 0.001**	−1.16	0.14	−8.38(35)	**< 0.001**
Familiarity^a^	0.56	0.09	6.07(740)	**< 0.001**	0.15	0.08	1.76(760)	0.08	−0.30	0.09	−3.35(716)	**< 0.001**
Time^b^	0.82	0.13	6.10(47)	**< 0.001**	0.38	0.17	2.24(33)	0.03	0.24	0.18	1.32(37)	0.20
Familiarity × Time interaction	−1.32	0.13	−10.13(740)	**< 0.001**	−0.60	0.12	−5.09(760)	**< 0.001**	0.27	0.13	2.16(716)	0.03

Significant *p* values (< 0.017) denoted in bold.

^a^Familiarity = trained/untrained.

^b^Time = T1/T2.

^c^The significance of fixed effects was assessed using *t*‐tests with Satterthwaite's approximation for degrees of freedom.

^d^Bonferroni correction was used. Adjusted alpha value was set to *α* = 0.017.

**FIGURE 5:**
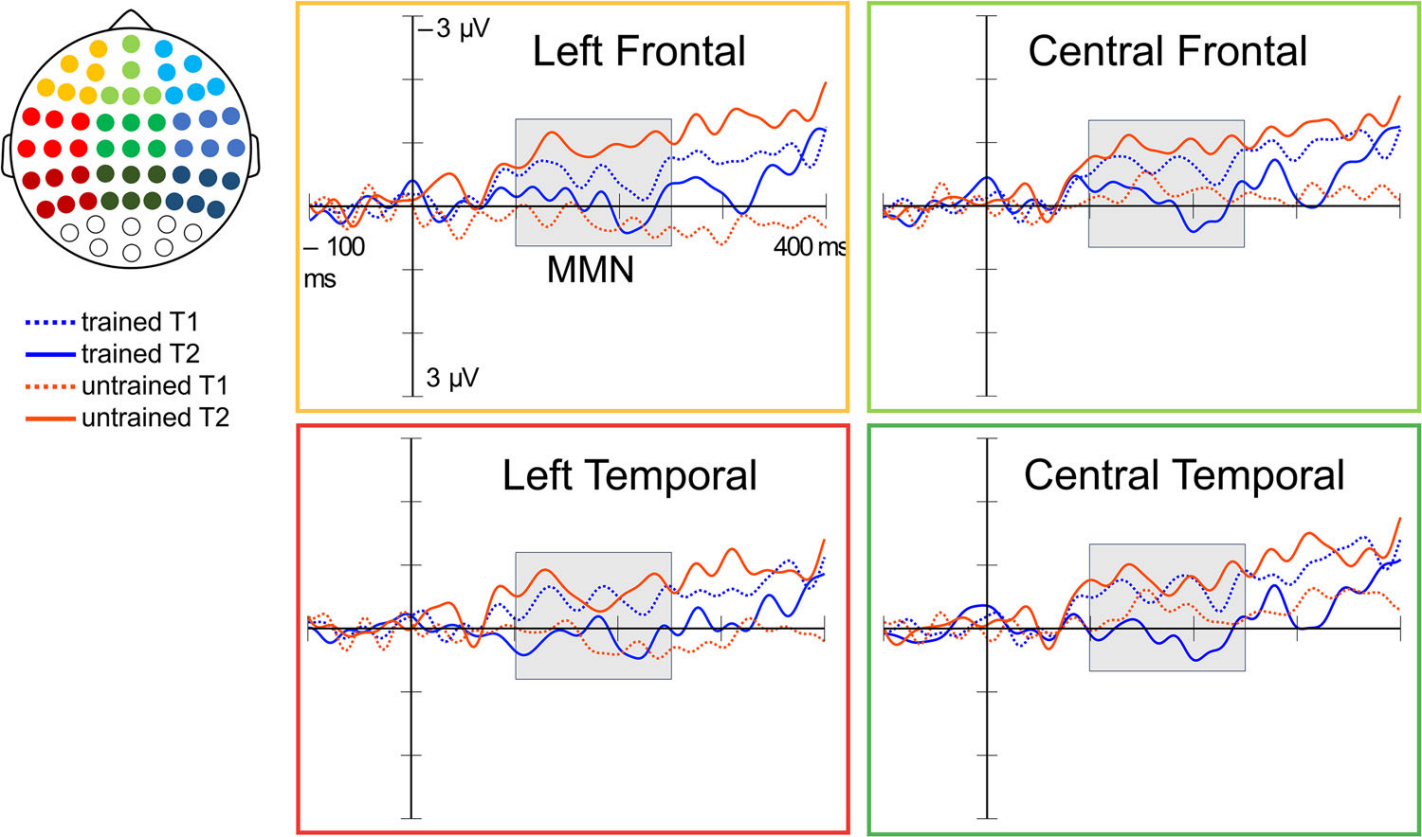
Mismatch negativity to phoneme deviants (deviant‐standard difference). MMN, mismatch negativity.

The direction and location of the Time × Familiarity interactions for phoneme and frequency MMNs were further explored with individual LMMs performed for each ROI (Table 3). For the phoneme MMN, the Time × Familiarity interaction was significant in four ROIs located in the left and central frontotemporal areas: left frontal (LF), central frontal (CF), left temporal (LT), and central temporal (CT). The model estimates for each ROI suggested a similar direction of change as the main model; MMN amplitude to the trained song showed model‐estimated decrease, while MMN amplitude to untrained song showed relative increase. The deviant‐standard difference waveforms and mean amplitudes of the MMN of these ROIs are shown in Figure 5. For the frequency MMN responses, none of the individual LMMs on each ROI showed significant effects (Table 3).

**TABLE 3:** Estimates of the interaction of Time and Familiarity in the individual linear mixed models on each ROI, and corresponding standard errors, *t* values, and *p* values.

		Phoneme deviants				Frequency deviants			
ROI		*β*	SE	*t*(df)^a^	*p* ^b^	*β*	SE	*t*(df)	*p* ^b^
Left frontal		−1.80	0.49	−3.70(69)	**< 0.001**	−0.30	0.55	−0.55	0.60
Central frontal		−1.7	0.48	−3.57(69)	**< 0.001**	−0.62	0.51	−1.22	0.23
Right frontal		−1.07	0.45	−2.38(69)	0.02	−0.62	0.44	−1.43	0.16
Left temporal		−1.58	0.42	−3.70(69)	**< 0.001**	−0.87	0.40	−2.17	0.03
Central temporal		−1.50	0.49	−3.04(69)	**0.003**	−0.52	0.51	−1.01	0.31
Right temporal		−0.96	0.38	−2.53(69)	0.01	−0.28	0.34	−0.82	0.42
Left parietal		−1.16	0.46	−2.50(69)	0.01	−0.81	0.38	−2.13	0.04
Central parietal		−1.13	0.50	−2.23(69)	0.03	−0.80	0.44	−1.84	0.07
Right parietal		−0.96	0.40	−2.41(69)	0.02	−0.58	0.35	−1.65	0.10

Significant *p* values (< 0.0056) denoted in bold.

Abbreviation: ROI, region of interest.

^a^The significance of fixed effects was assessed using *t*‐tests with Satterthwaite's approximation for degrees of freedom.

^b^Bonferroni correction was used. Adjusted alpha value was set to *α* = 0.0056.

Post hoc *t*‐tests (Table 4) for the phoneme MMN response showed a significant amplitude decrease to the trained song in the CF, LT, and CT from T1 to T2 and, conversely, a significant amplitude increase to the untrained song in the LF, CF, LT, and CT from T1 to T2.

**TABLE 4:** Mean amplitudes (µV) of the phoneme MMN before (T1) and after (T2) the intervention.

	ROI	MMN T1	MMN T2	*t*(23)	*p*
Trained	Left frontal	−1.81	−1.40	1.71	0.10
	Central frontal	−2.03	−1.39	2.58	**0.02**
	Left temporal	−1.69	−1.06	2.41	**0.02**
	Central temporal	−1.98	−1.21	2.81	**0.01**
Untrained	Left frontal	−0.94	−2.34	−3.84	**< 0.001**
	Central frontal	−1.29	−2.35	−3.10	**0.005**
	Left temporal	−0.89	−1.83	−3.44	**0.002**
	Central temporal	−1.40	−2.12	−2.50	**0.02**

Significant *p* values (< 0.05) denoted in bold.

Abbreviations: MMN, mismatch negativity; ROI, region of interest.

### 3.2 Correlations Between MMN Responses and Communication Abilities and Verbal Learning

Changes induced by the intervention in the Responsive speech and Communication indices have already been thoroughly reported in Siponkoski et al. [42], in which significant improvement in both indices following the singing intervention was observed. Pre‐ to postintervention scores from the verbal learning task were analyzed using paired sample *t*‐tests. Significant improvement was found in the learning task of the trained song from T1 (M = 86.7, SD = 13.6) to T2 (M = 93.8, SD = 10.2) (*t*(20) = 2.96, *p* = 0.008). No significant changes were seen in the score of the learning task of the untrained song.

Correlation analyses were performed to determine whether the observed training‐induced changes in phoneme MMN response in the four ROIs (LF, CF, LT, CT) were linked to improved verbal learning of the song lyrics as well as improved Communication and Responsive speech index scores from T1 to T2. Positive correlations were observed between improvement in Communication index and decrease of MMN amplitude in CT (*r* = 0.44, *p* = 0.03) and between the improvement in Responsive speech index and decrease of MMN amplitude in LT (*r* = 0.44, *p* = 0.03) and CT (*r* = 0.42, *p* = 0.04; Table 5), but after a Bonferroni correction for multiple comparisons, none of these correlations remained significant. There were no significant correlations between changes in phoneme MMN to the trained song and verbal learning or between changes in phoneme MMN to the untrained song and any behavioral outcome.

**TABLE 5:** Pearson correlations between changes seen in the MMN response, and changes seen in the linguistic outcome measures.

	ROI	Communication index	Responsive speech index	Learning task (*trained* song)	Learning task (*untrained* song)
Trained	Left frontal	0.34 (0.11)	0.34 (0.10)	−0.15 (.53)	0.38 (0.08)
	Central frontal	0.25 (0.24)	0.39 (0.06)	−0.04 (0.86)	0.23 (0.30)
	Left temporal	0.16 (0.47)	0.44 (0.03)	−0.21 (0.37)	0.02 (0.94)
	Central temporal	0.44 (0.03)	0.42 (0.04)	0.04 (0.86)	0.05 (0.83)
Untrained	Left frontal	−0.13 (0.56)	−0.12 (0.57)	0.04 (0.85)	0.03 (0.88)
	Central frontal	−0.22 (0.32)	−0.12 (0.56)	0.05 (0.82)	−0.02 (0.93)
	Left temporal	−0.16 (0.45)	−0.09 (0.67)	0.13 (0.59)	0.07 (0.75)
	Central temporal	0.11 (0.63)	−0.08 (0.71)	−0.03 (0.91)	0.05 (0.82)

Two‐tailed *p* values are shown in brackets. The adjusted (Bonferroni‐corrected) alpha value was set to *α* = 0.0016.

Abbreviation: ROI, region of interest.

## 4 Discussion

This study set out to explore the effects of a multicomponent singing intervention on behavioral and neural indices of novel song learning in PWA, as reflected by verbal production performance and the MMN responses to trained versus untrained songs. Following the intervention, the PWA showed enhanced verbal production of the trained song, coupled with changes in the MMN amplitudes for phoneme and frequency deviants. The model estimates indicated that MMN amplitude to the trained song decreased for phoneme and frequency deviants, while responses to the untrained song increased for phoneme deviants and remained stable for frequency deviants. These results indicate feature‐specific learning induced by the intervention, aligning with earlier findings that rehabilitation can influence the MMN response in PWA [29, 30]. Previous research has primarily focused on different forms of speech and language therapy, demonstrating their enhancing effects on the MMN response. The results of this study expand upon this knowledge by suggesting that also singing‐based rehabilitation can induce neural learning effects in PWA.

For phoneme deviants, significant intervention effects were seen in four frontotemporal ROIs, focusing on the left hemisphere. This finding of lateralization of the intervention effect for phoneme deviants is in line with previous research in which speech and language therapy has been found to induce left‐lateralized changes in the MMN to linguistic stimuli [29, 30, 60], and similar lateralization has been found for other language‐related ERPs [61]. Given that PWA typically show impaired linguistic MMN responses, especially in the left hemisphere [19, 20, 21, 22, 23, 24, 25, 26, 27, 28], these findings suggest that rehabilitation targeting speech outcomes can help restore the left‐lateralized pattern of linguistic‐auditory processing. In contrast, frequency deviants did not show significant intervention effects in individual ROIs. This may reflect the fact that the Time × Familiarity interactions were somewhat smaller across ROIs for the frequency MMN than the phoneme MMN and the current sample was not sufficiently large to detect these at ROI level. Alternatively, given that phoneme changes have been reported to induce larger and more left‐lateralized MMN responses than frequency changes [62, 63, 64] and show more impairment in aphasia [19, 22, 24, 27], it is also possible that the changes induced by the intervention in PWA are more pronounced and localized for phonemic deviants. This claim is, however, largely hypothetical and should be further explored empirically.

This study also aimed to investigate whether the effect of the intervention is specific to the trained song material or also generalizes to untrained song material. The results showed that following the intervention the MMN response to phoneme deviants decreased for the trained song and increased for the untrained song. In previous aphasia studies, increases (rather than decreases) of the MMN induced by speech and language therapy has been linked to improvement in language performance [30] or no correlation has been observed [29]; however, neither of these studies used EEG stimuli specific to the material used in the speech and language therapy, which makes their findings difficult to compare to ours in which the EEG stimuli were directly taken from the intervention material. While our results may seem contrary to the commonly held view that training generally leads to larger MMNs, they could be interpreted within the predictive coding framework [2, 65]. Extensive exposure to the trained song, including its numerous renditions produced by different PWA participants, with varying phonemic proficiency, during the singing intervention might make deviations from the original song less unpredictable to the participant and thus lead to more efficient adaptation and reduction of prediction errors (and smaller MMN) to phoneme changes over time. Conversely, the increased amplitude of the MMN to the phonemic deviants in the untrained song suggests heightened sensitivity to deviants in the novel material, resulting in increased prediction errors (and larger MMN). This could also be partially due to increased orienting of attention toward the phonemic features of the novel song, as attention has been shown to modulate the MMN response [66, 67, 68], but this remains speculative given the passive nature of the task.

Language performance and verbal learning (as indicated by increased number of correctly produced syllables in the trained song from T1 to T2) of the PWA improved following the intervention, suggesting that PWA are able to learn novel song lyrics with training. Following the intervention, the attenuation of the phoneme MMN response to the trained song in the left and central temporal regions was linked to improved language performance, particularly in everyday communication and cued verbal expression, which were the two main positive outcomes of the RCT [42]. However, given that these correlations did not survive the multiple comparisons correction, the results should be considered tentative and interpreted with caution. No connections were found between the enhancement of the MMN to the untrained song and language performance, suggesting that change only in the neural linguistic processing of the trained material may potentially be associated with treatment‐induced gains in language outcome.

Surprisingly, the attenuation of the phoneme MMN response to the trained song was not directly linked to the verbal learning of the trained song. Therefore, it is not clear to what extent the neural encoding of the trained material and its verbal production are associated. The lack of significant correlation may be due to the relatively small sample size, variability in aphasia severity, and lesion characteristics. On the other hand, based on current neurocognitive models of word learning, it is possible that these processes are governed by partially overlapping but distinct learning systems, with verbal production being linked more to the auditory‐motor dorsal system, which sustains the initial phonological processing and motor rehearsal of word representations, and neural encoding being linked more to the auditory‐perceptual ventral system, which combines phonological with semantic information [71, 72]. Previous behavioral studies have shown that word learning ability can be preserved in aphasia and is associated with a positive treatment response of speech and language therapy [69, 70, 71], but for singing interventions, more research is needed to specify this connection.

Although the current study provides insights into the effects of a multicomponent singing intervention on learning of novel song material in aphasia, there are some methodological limitations and several avenues for further exploration to be considered. The moderate sample size in this study might restrict the generalizability of the findings and decrease the visibility of weaker effects. There was also notable variation in the participants’ aphasia severity and the amount of time that had passed since their stroke, which could potentially have an impact on their response to the intervention. As expected, there was also considerable variation in the baseline amplitudes of the MMN responses and the magnitude of change following the intervention. Additionally, while EEG provides valuable insights into neural processing with high temporal resolution, its spatial resolution is limited. Methods such as magnetoencephalography (MEG) could allow for more detailed source localization and provide a clearer picture of how the neural networks underlying the MMN response may be shaped by the intervention. Finally, the longevity of the observed changes is unknown, as the current study focused on a rather short time period (pre‐ to postintervention) and did not analyze long‐term effects.

In conclusion, the results of this study show that during a 16‐week singing‐based rehabilitation program, PWA are able to learn novel songs, as indicated by improved verbal production of the trained song and more efficient neural adaptation to phoneme changes to the trained song and also heightened neural sensitivity to these deviants in new (untrained) song material, specifically in left frontotemporal areas. The clinical relevance of this preserved neural learning capacity in mediating the efficacy of singing‐based rehabilitation needs to be further explored.

## Author Contributions

Anni Pitkäniemi, Sini‐Tuuli Siponkoski, Sari Laitinen, Essi‐Reetta Särkämö, and Teppo Särkämö conceptualized the study. Emmi Pentikäinen, Anni Pitkäniemi, Sini‐Tuuli Siponkoski, Tommi Makkonen, Jaakko Kauramäki, and Teppo Särkämö planned the methodology. Teppo Särkämö handled project administration and funding acquisition. Aleksi J. Sihvonen and Teppo Särkämö supervised the work. Emmi Pentikäinen, Anni Pitkäniemi, and Sini‐Tuuli Siponkoski collected the data. Sari Laitinen and Essi‐Reetta Särkämö wrote the novel songs and led the singing intervention. Emma Oksanen, Emmi Pentikäinen, Tommi Makkonen, Jaakko Kauramäki, and Teppo Särkämö analyzed the data. Emma Oksanen wrote the original manuscript and performed visualization. All the authors reviewed, edited, and approved the final manuscript.

## Conflicts of Interest

The authors declare no conflicts of interest.

## Supporting information




**Supporting Information Figure S1**: nyas70087‐sup‐0001‐FigureS1.png

**Supporting Information Table S1**: nyas70087‐sup‐0002‐TableS1.docx

## Data Availability

The data that support the findings of this study are available from the corresponding author upon reasonable request.

## References

[1] A. S. Bregman , Auditory Scene Analysis: The Perceptual Organization of Sound (MIT Press, 1990).

[2] K. Friston , “A Theory of Cortical Responses,” Philosophical Transactions of the Royal Society B: Biological Sciences 360, no. 1456 (2005): 815–836, 10.1098/rstb.2005.1622. PMC1569488 15937014

[3] K. Fitzgerald and J. Todd , “Making Sense of Mismatch Negativity,” Frontiers in Psychiatry 11 (2020): 468, 10.3389/fpsyt.2020.00468. 32595529 PMC7300203

[4] R. Näätänen , “The Perception of Speech Sounds by the human Brain as Reflected by the Mismatch Negativity (MMN) and Its Magnetic Equivalent (MMNm),” Psychophysiology 38, no. 1 (2001): 1–21, 10.1111/1469-8986.3810001. 11321610

[5] R. Näätänen , A. W. K. Gaillard , and S. Mäntysalo , “Early Selective‐Attention Effect on Evoked Potential Reinterpreted,” Acta Psychologica 42, no. 4 (1978): 313–329, 10.1016/0001-6918(78)90006-9. 685709

[6] R. Näätänen , T. Jacobsen , and I. Winkler , “Memory‐Based or Afferent Processes in Mismatch Negativity (MMN): A Review of the Evidence,” Psychophysiology 42, no. 1 (2005): 25–32, 10.1111/j.1469-8986.2005.00256.x. 15720578

[7] G. Dehaene‐Lambertz , “Electrophysiological Correlates of Categorical Phoneme Perception in Adults,” Neuroreport 8, no. 4 (1997): 919–924. 9141065 10.1097/00001756-199703030-00021

[8] R. Näätänen , A. Lehtokoski , M. Lennes , et al., “Language‐Specific Phoneme Representations Revealed by Electric and Magnetic Brain Responses,” Nature 385, no. 6615 (1997): 432–434, 10.1038/385432a0. 9009189

[9] K. Alho , M. Tervaniemi , M. Huotilainen , et al., “Processing of Complex Sounds in the Human Auditory Cortex as Revealed by Magnetic Brain Responses,” Psychophysiology 33, no. 4 (1996): 369–375, 10.1111/j.1469-8986.1996.tb01061.x. 8753936

[10] M. Tervaniemi , A. Kujala , K. Alho , J. Virtanen , R. J. Ilmoniemi , and R. Näätänen , “Functional Specialization of the Human Auditory Cortex in Processing Phonetic and Musical Sounds: A Magnetoencephalographic (MEG) Study,” Neuroimage 9, no. 3 (1999): 330–336, 10.1006/nimg.1999.0405. 10075902

[11] K. Alho , “Cerebral Generators of Mismatch Negativity (MMN) and Its Magnetic Counterpart (MMNm) Elicited by Sound Changes,” Ear and Hearing 16, no. 1 (1995): 38–51, 10.1097/00003446-199502000-00004. 7774768

[12] R. Näätänen , P. Paavilainen , T. Rinne , and K. Alho , “The Mismatch Negativity (MMN) in Basic Research of Central Auditory Processing: A Review,” Clinical Neurophysiology 118, no. 12 (2007): 2544–2590, 10.1016/j.clinph.2007.04.026. 17931964

[13] F. Pulvermüller , Y. Shtyrov , and R. Ilmoniemi , “Spatiotemporal Dynamics of Neural Language Processing: An MEG Study Using Minimum‐Norm Current Estimates,” Neuroimage 20, no. 2 (2003): 1020–1025, 10.1016/S1053-8119(03)00356-2. 14568471

[14] Y. Shtyrov , E. Pihko , and F. Pulvermüller , “Determinants of Dominance: Is Language Laterality Explained by Physical or Linguistic Features of Speech?,” Neuroimage 27, no. 1 (2005): 37–47, 10.1016/j.neuroimage.2005.02.003. 16023039

[15] T. L. Van Zuijen , V. L. Simoens , P. Paavilainen , R. Näätänen , and M. Tervaniemi , “Implicit, Intuitive, and Explicit Knowledge of Abstract Regularities in a Sound Sequence: An Event‐Related Brain Potential Study,” Journal of Cognitive Neuroscience 18, no. 8 (2006): 1292–1303, 10.1162/jocn.2006.18.8.1292. 16859415

[16] H. L. Flowers , S. A. Skoretz , F. L. Silver , et al., “Poststroke Aphasia Frequency, Recovery, and Outcomes: A Systematic Review and Meta‐Analysis,” Archives of Physical Medicine and Rehabilitation 97, no. 12 (2016): 2188–2201.e8, 10.1016/j.apmr.2016.03.006. 27063364

[17] P. M. Pedersen , H. Stig Jørgensen , H. Nakayama , H. O. Raaschou , and T. S. Olsen , “Aphasia in Acute Stroke: Incidence, Determinants, and Recovery,” Annals of Neurology 38, no. 4 (1995): 659–666, 10.1002/ana.410380416. 7574464

[18] P. M. Pedersen , K. Vinter , and T. S. Olsen , “Aphasia After Stroke: Type, Severity and Prognosis,” Cerebrovascular Diseases 17, no. 1 (2004): 35–43, 10.1159/000073896. 14530636

[19] O. Aaltonen , J. Tuomainen , M. Laine , and P. Niemi , “Cortical Differences in Tonal Versus Vowel Processing as Revealed by an ERP Component Called Mismatch Negativity (MMN),” Brain and Language 44, no. 2 (1993): 139–152, 10.1006/brln.1993.1009. 8428308

[20] A. Aerts , P. Van Mierlo , R. J. Hartsuiker , P. Santens , and M. De Letter , “Neurophysiological Sensitivity for Impaired Phonological Processing in the Acute Stage of Aphasia,” Brain and Language 149 (2015): 84–96, 10.1016/j.bandl.2015.07.001. 26197257

[21] F. Becker and I. Reinvang , “Mismatch Negativity Elicited by Tones and Speech Sounds: Changed Topographical Distribution in Aphasia,” Brain and Language 100, no. 1 (2007): 69–78, 10.1016/j.bandl.2006.09.004. 17069882

[22] V. Csépe , J. Osman‐Sági , M. Molnár , and M. Gósy , “Impaired Speech Perception in Aphasic Patients: Event‐Related Potential and Neuropsychological Assessment,” Neuropsychologia 39, no. 11 (2001): 1194–1208, 10.1016/S0028-3932(01)00052-5. 11527557

[23] T.‐M. Ilvonen , T. Kujala , A. Kiesiläinen , et al., “Auditory Discrimination After Left‐Hemisphere Stroke: A Mismatch Negativity Follow‐Up Study,” Stroke 34, no. 7 (2003): 1746–1751, 10.1161/01.STR.0000078836.26328.3B. 12817100

[24] T.‐M. Ilvonen , T. Kujala , H. Kozou , et al., “The Processing of Speech and Non‐Speech Sounds in Aphasic Patients as Reflected by the Mismatch Negativity (MMN),” Neuroscience Letters 366, no. 3 (2004): 235–240, 10.1016/j.neulet.2004.05.024. 15288425

[25] T.‐M. Ilvonen , T. Kujala , M. Tervaniemi , O. Salonen , R. Näätänen , and E. Pekkonen , “The Processing of Sound Duration After Left Hemisphere Stroke: Event‐Related Potential and Behavioral Evidence,” Psychophysiology 38, no. 4 (2001): 622–628, 10.1111/1469-8986.3840622. 11446575

[26] C. Pettigrew , B. Murdoch , J. Kei , C. Ponton , P. Alku , and H. Chenery , “The Mismatch Negativity (MMN) Response to Complex Tones and Spoken Words in Individuals With Aphasia,” Aphasiology 19, no. 2 (2005): 131–163, 10.1080/02687030444000642.

[27] H. Robson , L. Cloutman , J. L. Keidel , K. Sage , M. Drakesmith , and S. Welbourne , “Mismatch Negativity (MMN) Reveals Inefficient Auditory Ventral Stream Function in Chronic Auditory Comprehension Impairments,” Cortex 59 (2014): 113–125, 10.1016/j.cortex.2014.07.009. 25173955

[28] S. Teki , G. R. Barnes , W. D. Penny , et al., “The Right Hemisphere Supports but Does Not Replace Left Hemisphere Auditory Function in Patients With Persisting Aphasia,” Brain 136, no. 6 (2013): 1901–1912, 10.1093/brain/awt087. 23715097 PMC3673464

[29] G. Lucchese , F. Pulvermüller , B. Stahl , F. R. Dreyer , and B. Mohr , “Therapy‐Induced Neuroplasticity of Language in Chronic Post Stroke Aphasia: A Mismatch Negativity Study of (A)Grammatical and Meaningful/Less Mini‐Constructions,” Frontiers in Human Neuroscience 10 (2017): 669, 10.3389/fnhum.2016.00669. 28111545 PMC5216683

[30] B. Mohr , L. J. MacGregor , S. Difrancesco , K. Harrington , F. Pulvermüller , and Y. Shtyrov , “Hemispheric Contributions to Language Reorganisation: An MEG Study of Neuroplasticity in Chronic Post Stroke Aphasia,” Neuropsychologia 93 (2016): 413–424, 10.1016/j.neuropsychologia.2016.04.006. 27063061

[31] J. K. Johnson and A. B. Graziano , “Some Early Cases of Aphasia and the Capacity to Sing,” Progress in Brain Research 216 (2015): 73–89, 10.1016/bs.pbr.2014.11.004. 25684286

[32] A. Racette , C. Bard , and I. Peretz , “Making Non‐Fluent Aphasics Speak: Sing Along,” Brain 129, no. 10 (2006): 2571–2584, 10.1093/brain/awl250. 16959816

[33] A. Yamadori , Y. Osumi , S. Masuhara , and M. Okubo , “Preservation of Singing in Broca's Aphasia,” Journal of Neurology, Neurosurgery & Psychiatry 40, no. 3 (1977): 221–224, 10.1136/jnnp.40.3.221. 886348 PMC492653

[34] K. J. Jeffries , J. B. Fritz , and A. R. Braun , “Words in Melody: An H215O PET Study of Brain Activation During Singing and Speaking,” Neuroreport 14, no. 5 (2003): 749–754, 10.1097/00001756-200304150-00018. 12692476

[35] E. Özdemir , A. Norton , and G. Schlaug , “Shared and Distinct Neural Correlates of Singing and Speaking,” Neuroimage 33, no. 2 (2006): 628–635, 10.1016/j.neuroimage.2006.07.013. 16956772

[36] D. W. Perry , R. J. Zatorre , M. Petrides , B. Alivisatos , E. Meyer , and A. C. Evans , “Localization of Cerebral Activity During Simple Singing,” Neuroreport 10, no. 18 (1999): 3979–3984. 10716244 10.1097/00001756-199912160-00046

[37] N. Martínez‐Molina , S. T. Siponkoski , A. Pitkäniemi , et al., “Neuroanatomical Correlates of Speech and Singing Production in Chronic Post‐Stroke Aphasia,” Brain Communications 4, no. 1 (2022): fcac001, 10.1093/braincomms/fcac001. 35174327 PMC8842683

[38] A. Pitkäniemi , T. Särkämö , S. T. Siponkoski , et al., “Hodological Organization of Spoken Language Production and Singing in the Human Brain,” Communications Biology 6, no. 1 (2023): 779, 10.1038/s42003-023-05152-y. 37495670 PMC10371982

[39] W. L. Magee , I. Clark , J. Tamplin , and J. Bradt , “Music Interventions for Acquired Brain Injury,” Cochrane Database of Systematic Reviews 2017, no. 1 (2017): CD006787, 10.1002/14651858.CD006787.pub3. PMC6464962 28103638

[40] T. Popescu , B. Stahl , B. M. Wiernik , et al., “Melodic Intonation Therapy for Aphasia: A Multi‐Level Meta‐Analysis of Randomized Controlled Trials and Individual Participant Data,” Annals of the New York Academy of Sciences 1516, no. 1 (2022): 76–84, 10.1111/nyas.14848. 35918503 PMC9804200

[41] A. J. Sihvonen , T. Särkämö , V. Leo , M. Tervaniemi , E. Altenmüller , and S. Soinila , “Music‐Based Interventions in Neurological Rehabilitation,” Lancet Neurology 16, no. 8 (2017): 648–660, 10.1016/S1474-4422(17)30168-0. 28663005

[42] S.‐T. Siponkoski , A. Pitkäniemi , S. Laitinen , et al., “Efficacy of a Multicomponent Singing Intervention on Communication and Psychosocial Functioning in Chronic Aphasia: A Randomized Controlled Crossover Trial,” Brain Communications 5, no. 1 (2022): fcac337, 10.1093/braincomms/fcac337. 36687394 PMC9847537

[43] J. Tamplin , F. A. Baker , B. Jones , A. Way , and S. Lee , “Stroke a Chord': The Effect of Singing in a Community Choir on Mood and Social Engagement for People Living With Aphasia Following a Stroke,” Neurorehabilitation 32, no. 4 (2013): 929–941, 10.3233/NRE-130916. 23867418

[44] A. Zumbansen , I. Peretz , C. Anglade , et al., “Effect of Choir Activity in the Rehabilitation of Aphasia: A Blind, Randomised, Controlled Pilot Study,” Aphasiology 31, no. 8 (2017): 879–900, 10.1080/02687038.2016.1227424.

[45] A. J. Sihvonen , A. Pitkäniemi , S.‐T. Siponkoski , et al., “Structural Neuroplasticity Effects of Singing in Chronic Aphasia,” Eneuro 11, no. 5 (2024): ENEURO.0408–23.2024, 10.1523/ENEURO.0408-23.2024. 38688718 PMC11091951

[46] H. Goodglass , E. Kaplan , and S. Weintraub , BDAE: The Boston Diagnostic Aphasia Examination (Lippincott Williams & Wilkins, 2001).

[47] A. Norton , L. Zipse , S. Marchina , and G. Schlaug , “Melodic Intonation Therapy: Shared Insights on How It Is Done and Why It Might Help,” Annals of the New York Academy of Sciences 1169, no. 1 (2009): 431–436, 10.1111/j.1749-6632.2009.04859.x. 19673819 PMC2780359

[48] J. Marco‐Pallarés , C. Grau , and G. Ruffini , “Combined ICA‐LORETA Analysis of Mismatch Negativity,” Neuroimage 25, no. 2 (2005): 471–477, 10.1016/j.neuroimage.2004.11.028. 15784426

[49] F. J. Hsiao , C. H. Cheng , K. K. Liao , and Y. Y. Lin , “Cortico‐Cortical Phase Synchrony in Auditory Mismatch Processing,” Biological Psychology 84, no. 2 (2010): 336–345, 10.1016/j.biopsycho.2010.03.019. 20380866

[50] C. Lappe , O. Steinsträter , and C. Pantev , “Rhythmic and Melodic Deviations in Musical Sequences Recruit Different Cortical Areas for Mismatch Detection,” Frontiers in Human Neuroscience 7 (2013): 260, 10.3389/fnhum.2013.00260. 23759929 PMC3675320

[51] T. Rinne , K. Alho , R. J. Ilmoniemi , J. Virtanen , and R. Näätänen , “Separate Time Behaviors of the Temporal and Frontal Mismatch Negativity Sources,” Neuroimage 12, no. 1 (2000): 14–19, 10.1006/nimg.2000.0591. 10875898

[52] Y. Criel , E. Depuydt , M. Miatton , P. Santens , P. van Mierlo , and M. De Letter , “Cortical Generators and Connections Underlying Phoneme Perception: A Mismatch Negativity and P300 Investigation,” Brain Topography 37, no. 6 (2024): 1089–1117, 10.1007/s10548-024-01065-z. 38958833

[53] T. E. Cope , L. E. Hughes , H. N. Phillips , et al., “Causal Evidence for the Multiple Demand Network in Change Detection: Auditory Mismatch Magnetoencephalography Across Focal Neurodegenerative Diseases,” Journal of Neuroscience 42, no. 15 (2022): 3197–3215, 10.1523/jneurosci.1622-21.2022. 35260433 PMC8994545

[54] F. Pulvermüller , B. Neininger , T. Elbert , et al., “Constraint‐Induced Therapy of Chronic Aphasia After Stroke,” Stroke 32, no. 7 (2001): 1621–1626, 10.1161/01.STR.32.7.1621. 11441210

[55] P. W. Duncan , R. K. Bode , S. Min Lai , and S. Perera , “Rasch Analysis of a New Stroke‐Specific Outcome Scale: The Stroke Impact Scale,” Archives of Physical Medicine and Rehabilitation 84, no. 7 (2003): 950–963, 10.1016/S0003-9993(03)00035-2. 12881816

[56] A. Kertesz , Western Aphasia Battery (Psychological Corporation, 1982).

[57] A. Stockert , M. Wawrzyniak , J. Klingbeil , et al., “Dynamics of Language Reorganization After Left Temporo‐Parietal and Frontal Stroke,” Brain 143, no. 3 (2020): 844–861, 10.1093/brain/awaa023. 32068789

[58] R Core Team , R: A Language and Environment for Statistical Computing [Computer Software] (R Foundation for Statistical Computing, 2022), https://www.r‐project.org/.

[59] D. Bates , M. Mächler , B. Bolker , and S. Walker , “Fitting Linear Mixed‐Effects Models Using lme4,” Journal of Statistical Software 67, no. 1 (2015): 1–48, 10.18637/jss.v067.i01.

[60] Z. V. Woodhead , J. Crinion , S. Teki , W. Penny , C. J. Price , and A. P. Leff , “Auditory Training Changes Temporal Lobe Connectivity in ‘Wernicke's Aphasia’: A Randomised Trial,” Journal of Neurology, Neurosurgery & Psychiatry 88, no. 7 (2017): 586–594, 10.1136/jnnp-2016-314621. 28259857 PMC5659142

[61] F. Pulvermüller , O. Hauk , K. Zohsel , B. Neininger , and B. Mohr , “Therapy‐Related Reorganization of Language in Both Hemispheres of Patients With Chronic Aphasia,” Neuroimage 28, no. 2 (2005): 481–489, 10.1016/j.neuroimage.2005.06.038. 16099176

[62] M. Jaramillo , T. Ilvonen , T. Kujala , P. Alku , M. Tervaniemi , and K. Alho , “Are Different Kinds of Acoustic Features Processed Differently for Speech and Non‐Speech Sounds?,” Cognitive Brain Research 12, no. 3 (2001): 459–466, 10.1016/s0926-6410(01)00081-7. 11689306

[63] S. Pakarinen , T. Teinonen , A. Shestakova , et al., “Fast Parametric Evaluation of Central Speech‐Sound Processing With Mismatch Negativity (MMN),” International Journal of Psychophysiology 87, no. 1 (2013): 103–110, 10.1016/j.ijpsycho.2012.11.010. 23201145

[64] D. L. Tang , R. Möttönen , S. S. Asaridou , and K. E. Watkins , “Asymmetry of Auditory‐Motor Speech Processing Is Determined by Language Experience,” Journal of Neuroscience 41, no. 5 (2021): 1059–1067, 10.1523/jneurosci.1977-20.2020. 33298537 PMC7880293

[65] M. I. Garrido , J. M. Kilner , K. E. Stephan , and K. J. Friston , “The Mismatch Negativity: A Review of Underlying Mechanisms,” Clinical Neurophysiology 120, no. 3 (2009): 453–463, 10.1016/j.clinph.2008.11.029. 19181570 PMC2671031

[66] E. S. Sussman , A. S. Bregman , W. J. Wang , and F. J. Khan , “Attentional Modulation of Electrophysiological Activity in Auditory Cortex for Unattended Sounds Within Multistream Auditory Environments,” Cognitive, Affective, & Behavioral Neuroscience 5, no. 1 (2005): 93–110, 10.3758/CABN.5.1.93. 15913011

[67] M. G. Woldorff , S. A. Hackley , and S. A. Hillyard , “The Effects of Channel‐Selective Attention on the Mismatch Negativity Wave Elicited by Deviant Tones,” Psychophysiology 28, no. 1 (1991): 30–42, 10.1111/j.1469-8986.1991.tb03384.x. 1886962

[68] M. G. Woldorff , S. A. Hillyard , C. C. Gallen , S. R. Hampson , and F. E. Bloom , “Magnetoencephalographic Recordings Demonstrate Attentional Modulation of Mismatch‐Related Neural Activity in Human Auditory Cortex,” Psychophysiology 35, no. 3 (1998): 283–292, 10.1017/S0048577298961601. 9564748

[69] J. Dignam , D. Copland , A. Rawlings , K. O'Brien , P. Burfein , and A. D. Rodriguez , “The Relationship Between Novel Word Learning and Anomia Treatment Success in Adults With Chronic Aphasia,” Neuropsychologia 81 (2016): 186–197, 10.1016/j.neuropsychologia.2015.12.026. 26724545

[70] L. M. Tuomiranta , E. Càmara , S. Froudist Walsh , et al., “Hidden Word Learning Capacity Through Orthography in Aphasia,” Cortex 50 (2014): 174–191, 10.1016/j.cortex.2013.10.003. 24262200 PMC8168370

[71] C. Peñaloza , N. Martin , M. Laine , and A. Rodríguez‐Fornells , “Language Learning in Aphasia: A Narrative Review and Critical Analysis of the Literature With Implications for Language Therapy,” Neuroscience & Biobehavioral Reviews 141 (2022): 104825, 10.1016/j.neubiorev.2022.104825. 35963544

[72] A. Rodríguez‐Fornells , T. Cunillera , A. Mestres‐Missé , and R. de Diego‐Balaguer , “Neurophysiological Mechanisms Involved in Language Learning in Adults,” Philosophical Transactions of the Royal Society of London Series B Biological Sciences 364, no. 1536 (2009): 3711–3735, 10.1098/rstb.2009.0130. 19933142 PMC2846313

