# Supplementary figures and images for: Training‐Induced Neural Enhancement of Novel Song Learning in Chronic Aphasia: EEG Study
Source: Ann N Y Acad Sci. 2025 Oct 13;1553(1):220–32. doi: 10.1111/nyas.70087 (PMC12645264; doi:10.1111/nyas.70087)

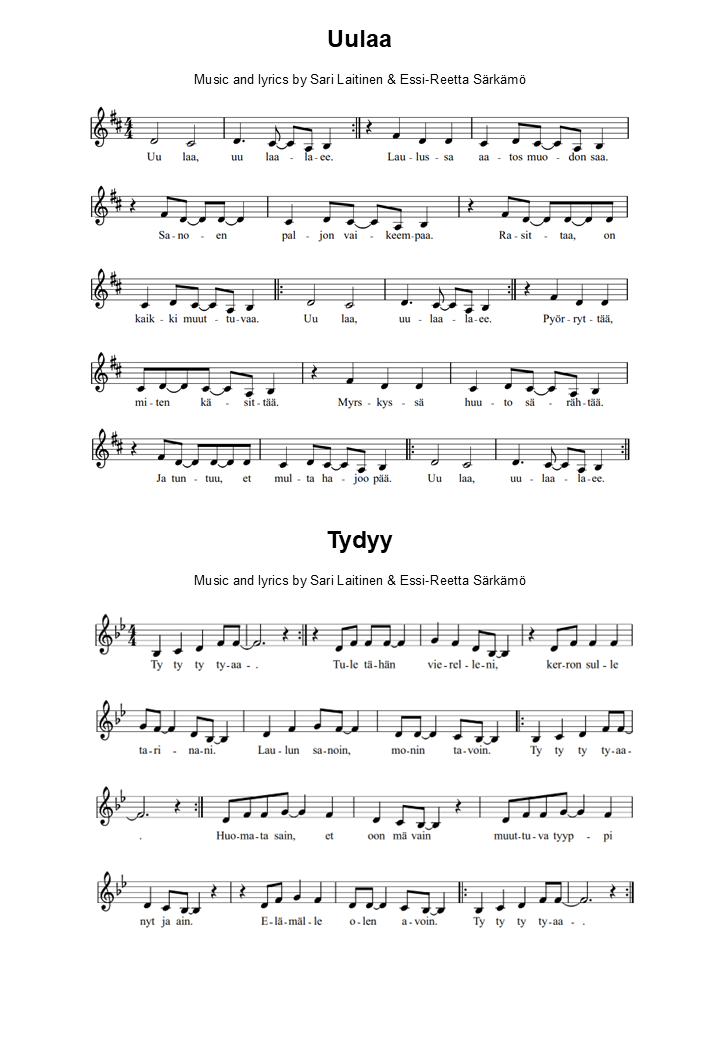

Supplement: Supplementary file 1 — Supporting Information Figure S1: nyas70087‐sup‐0001‐FigureS1.png [file NYAS-1553-220-s002.png]
