# Supplementary material for: Training‐Induced Neural Enhancement of Novel Song Learning in Chronic Aphasia: EEG Study
Source: Ann N Y Acad Sci. 2025 Oct 13;1553(1):220–32. doi: 10.1111/nyas.70087 (PMC12645264; doi:10.1111/nyas.70087)
Supplement: Supplementary file 2 — Supporting Information Table S1: nyas70087‐sup‐0002‐TableS1.docx [file NYAS-1553-220-s001.docx]

**SUPPORTING Table S1. Degrees of freedom, F-values and p-values for the main effects of the repeated-measures ANOVAs for phoneme, frequency and duration deviants at T1.**

|  | **Effect** | **df** | **F** | **p** |
| --- | --- | --- | --- | --- |
| Phoneme | Group | 22 | 0.09 | .77 |
|  | Song | 22 | 0.94 | .34 |
| Frequency | Group | 22 | 1.76 | .20 |
|  | Song | 22 | 0.00 | .98 |
| Duration | Group | 22 | 2.23 | .15 |
|  | Song | 22 | 0.12 | .74 |
